# Vitamin D pathway-related gene polymorphisms and their association with metabolic diseases: A literature review

**DOI:** 10.1007/s40200-020-00561-w

**Published:** 2020-06-16

**Authors:** Buthaina E. Alathari, Aji A. Sabta, Chinnappan A. Kalpana, Karani Santhanakrishnan Vimaleswaran

**Affiliations:** 1 Hugh Sinclair Unit of Human Nutrition, Department of Food and Nutritional Sciences, University of Reading, PO Box 226, Whiteknights, Reading, RG6 6AP UK; 2 Department of Food Science and Nutrition, Faculty of Health Sciences, The Public Authority for Applied Education and Training, P.O. Box 14281, AlFaiha , 72853 Kuwait; 3 Department of Nutrition, Faculty of Health Sciences, University Alma Ata, Yogyakarta, 55183 Indonesia; 4 Postgraduate Biomedical Science Department, Faculty of Medicine, Andalas University, West Sumatra , 25172 Indonesia; 5 Avinashilingam Institute for Home Science and Higher Education for Women, Coimbatore, Tamil Nadu India

**Keywords:** Vitamin D, 25(OH)D, Genetics of vitamin D, SNPs, Obesity, Type 2 diabetes

## Abstract

**Purpose:**

Given that the relationship between vitamin D status and metabolic diseases such as obesity and type 2 diabetes (T2D) remains unclear, this review will focus on the genetic associations, which are less prone to confounding, between vitamin D-related single nucleotide polymorphisms (SNPs) and metabolic diseases.

**Methods:**

A literature search of relevant articles was performed on PubMed up to December 2019. Those articles that had examined the association of vitamin D-related SNPs with obesity and/or T2D were included. Two reviewers independently evaluated the eligibility for the inclusion criteria and extracted the data. In total, 73 articles were included in this review.

**Results:**

There is a lack of research focusing on the association of vitamin D synthesis-related genes with obesity and T2D; however, the limited available research, although inconsistent, is suggestive of a protective effect on T2D risk. While there are several studies that investigated the vitamin D metabolism-related SNPs, the research focusing on vitamin D activation, catabolism and transport genes is limited. Studies on *CYP27B1*, *CYP24A1* and *GC* genes demonstrated a lack of association with obesity and T2D in Europeans; however, significant associations with T2D were found in South Asians. *VDR* gene SNPs have been extensively researched; in particular, the focus has been mainly on BsmI (rs1544410), TaqI (rs731236), ApaI (rs7975232) and FokI (rs2228570) SNPs. Even though the association between *VDR* SNPs and metabolic diseases remain inconsistent, some positive associations showing potential effects on obesity and T2D in specific ethnic groups were identified.

**Conclusions:**

Overall, this literature review suggests that ethnic-specific genetic associations are involved. Further research utilizing large studies is necessary to better understand these ethnic-specific genetic associations between vitamin D deficiency and metabolic diseases.

## Introduction

Vitamin D is a fat-soluble vitamin and a secosteroid prohormone that plays a crucial role in bone mineralization through the absorption and regulation of calcium and phosphate levels [1]. The vitamin D endocrine system regulates calcium homoeostasis and a range of physiological functions such as cell growth, proliferation, differentiation, immune function, inflammation, and apoptosis [2]. A broad spectrum of diseases has been related to vitamin D deficiency and research, to date, suggests that vitamin D deficiency is a marker of ill health with effective connection to all-cause mortality, obesity, diabetes, cardiovascular risk, hypertension, dyslipidaemia, multiple sclerosis, Alzheimer, and some types of cancer [3–7]. However, causality is yet to be proven for any disease that is associated with vitamin D deficiency.

**Fig. 1:**
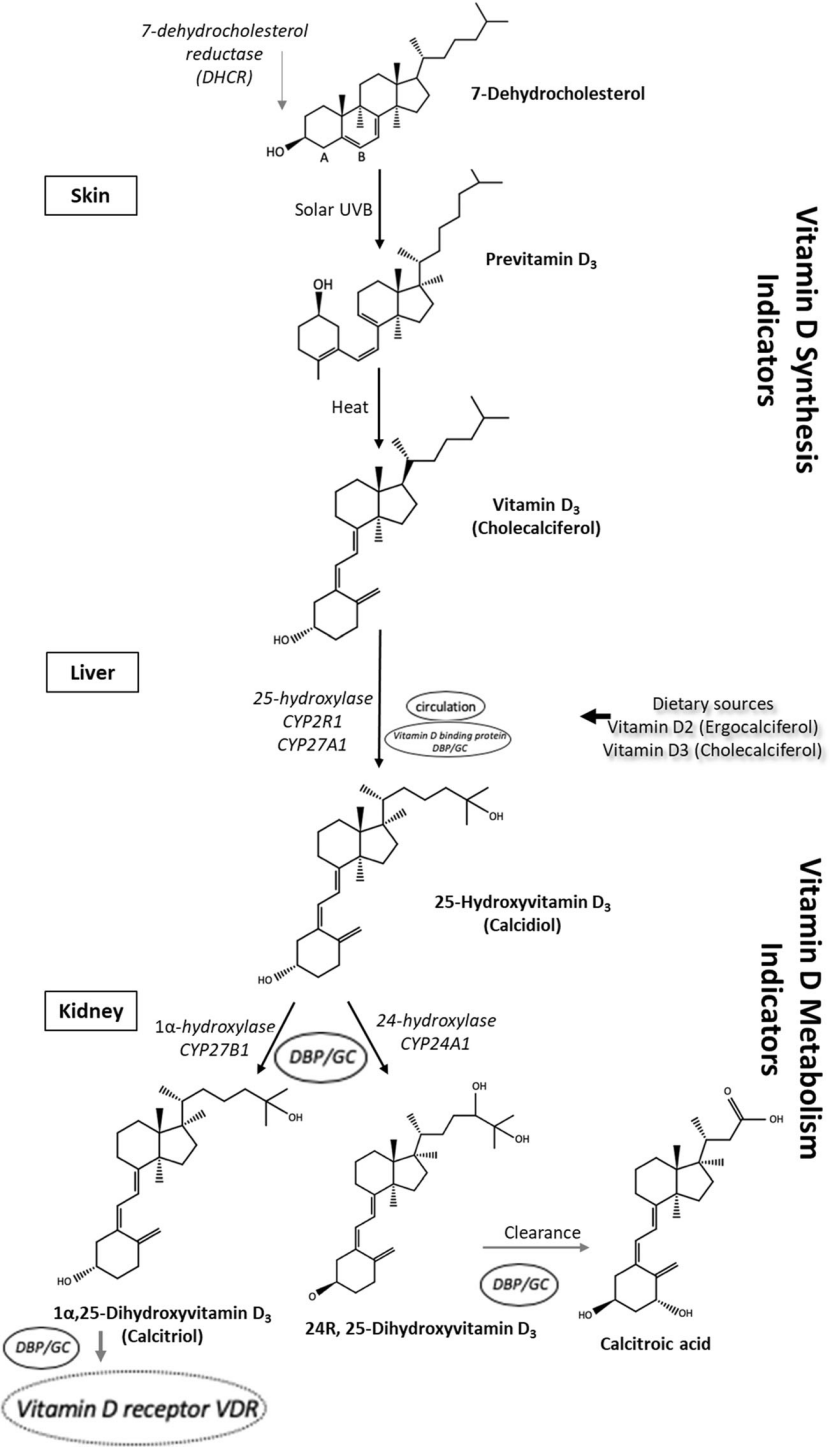
Vitamin D synthesis and metabolism. Upon exposure to Ultraviolet-B (290–315 nm wavelength) skin irradiation, 7-dehydrocholesterol produces pre-vitamin D3, by breaking the C (9–10) bond at the B ring, which then undergoes a thermally induced rearrangement to form vitamin D_3_. Vitamin D can also enter the body from dietary sources in two forms: vitamin D_3_ (cholecalciferol) from fish, eggs, fortified milk and supplements and vitamin D_2_ (ergocalciferol) from mushrooms and yeast. Once transported to the liver, vitamin D is hydroxylated to 25(OH)D (calcidiol) by 25-hydroxylase enzymes (*CYP2R1* & *CYP27A1*). In the kidneys, 25(OH)D is further hydroxylated by two enzymes to activate or inactivate vitamin D. For activation, the 1α-hydroxylase enzyme (*CYP27B1*) converts 25(OH)D to 1α,25-dihydroxyvitamin D (calcitriol), which is transported by vitamin D binding protein (DBP/GC). Finally, Calcitriol binds to vitamin D receptor (*VDR*) to perform its biological function. For inactivation, the 24-hydroxylase enzyme (*CYP24A1*) catabolizes 25(OH)D to 24,25-dihydroxy vitamin D. Control of metabolism of vitamin D is exerted primarily by biliary excretion

Vitamin D_3_ (cholecalciferol) is the natural form of vitamin D and the body can synthesise it in the skin in response to the sunlight exposure. Ultraviolet-B (UVB) (290–315 nm wavelength) skin irradiation initiates the photochemical conversion of 7DHC (provitamin D_3_) to previtamin D_3_ by breaking the 9,10 carbon-carbon bond, which is then quickly thermally isomerized to vitamin D_3_. Diet is another source of vitamin D_3_, which can be obtained from animal foods such as oily fish, egg yolk, liver, butter, fortified milk and cheese. Vitamin D_2_ (ergocalciferol) originates from conversion of a plant sterol, ergosterol, and is solely obtained from the diet which includes plant-sourced foods such as yeast and mushrooms [8, 9]. Vitamin D_3_ has a superior bioavailability than vitamin D_2_; nonetheless, they both go through the same metabolic pathway to produce the active hormonal forms [4, 10]. Vitamin D is biologically inert and has to undergo hydroxylation twice before it can perform its physiological functions. Vitamin D binding protein (DBP/GC) is the key transport protein, which binds over 85% of the circulating 25(OH)D and vitamin D metabolites, and it transports these metabolites to target cells. In the liver, vitamin D (cholecalciferol and ergocalciferol) is converted by the enzyme 25-hydroxylase (*CYP2R1*) into 25-hydroxyvitamin D [25(OH)D], also known as calcidiol, which is the primary circulating form of vitamin D. Subsequently, the kidney, acting as an endocrine gland, converts 25(OH)D by the action of the enzyme 1α-hydroxylase (*CYB27B1*) to the active hormonal form 1α, 25-dihydroxyvitamin D [1,25(OH)_2_D], also known as calcitriol, which then binds to *VDR* and regulates calcium homeostasis and bone metabolism (Fig. 1). The *VDR*, a member of the nuclear receptor family, is a receptor specific to vitamin D through which vitamin D exerts its function, and it has been discovered in a multitude of cell membranes of tissues that have no musculoskeletal function; this implies the involvement of vitamin D in various extra-skeletal biological functions [3, 4, 6].

Heritability of vitamin D deficiency has been reported by twin and family studies to range between 20–85% [11]. Although there is a great variation in the estimation of the heritability results, they do show that genetic factors play a role in circulating serum 25(OH)D levels. Candidate gene studies have reported several single nucleotide polymorphisms (SNPs) related to serum 25(OH)D levels mainly with genes that are involved in synthesis and metabolism of vitamin D such as *DHCR7*, *CYP2R1*, *CYP27B1*, *CYP24A1, DBP/GC*, *VDR* [12]. Genome wide association studies (GWAS) have confirmed the association between genetic polymorphisms in the genes such as *GC*, *DHCR7*, *CYP2R1* and *CYP24A1* and 25(OH)D concentrations [11].

There exists a plethora of studies that have reported association of genetic variants with low vitamin D levels and a wide spectrum of associated diseases [13–16]. This article aims to evaluate the results of the associations between vitamin D-related genetic variants and metabolic diseases such as obesity and type 2 diabetes (T2D). Understanding the possible underlying genetic factors of vitamin D metabolism will lead to an increased understanding of the biological mechanisms underlying vitamin D deficiency and its effects on metabolic diseases.

## Methods and materials

### Study identification

To review published research articles relevant to the topic, a literature search of PubMed (National Library of Medicine) https://www.ncbi.nlm.nih.gov/pubmed/ was performed up to December 2019. The following key terms were used to search for research articles: “vitamin D genetics and diabetes” (n = 543), “vitamin D genetics and obesity” (n = 202), “vitamin D gene polymorphisms and diabetes” (n = 308), “vitamin D gene polymorphisms and obesity” (n = 85), “Genetic variants of vitamin D and diabetes” (n = 79), “Genetic variants of vitamin D and obesity” (n = 31), “vitamin D SNPs and diabetes” (n = 150), “vitamin D SNPs and obesity” (n = 57). As a result of all the search combinations, a total of 1,455 articles were obtained. Citations from relevant papers and review papers were examined to identify additional relevant articles for inclusion.

### Study selection

Any study that was published in PubMed and written in English was included. Only genetic association studies examining the association of vitamin D-related SNPs with diabetes and/or obesity were included. Studies were excluded if they were (1) animal studies; (2) studies in pregnant women; (3) studies on humans identified with disease other than metabolic diseases; (4) randomized controlled trials; (5) gene-vitamin D interaction studies, (6) haplotype studies, (7) studies with outcome as serum 25(OH)D, bone disease, metabolic syndrome, type 1 diabetes, diabetic complications or any other disease except for obesity and T2D.

**Fig. 2:**
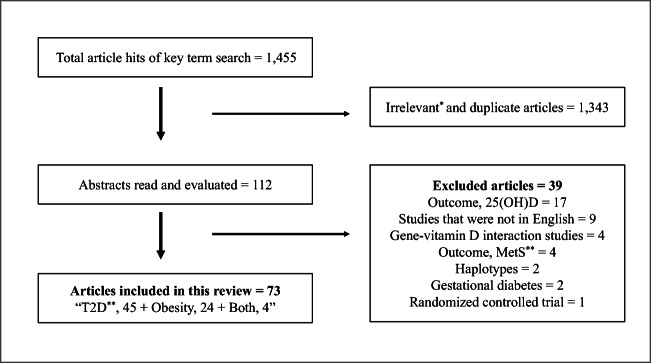
Flow chart describing the literature search and the steps involved in selecting the articles. ^*^Irrelevant articles are those that did not meet the inclusion criteria. ^**^MetS: Metabolic Syndrome; T2D: Type 2 diabetes

The article titles were reviewed to eliminate duplication and relevant papers were chosen (n = 112). Abstracts of the chosen articles were read to further determine their relevance to our topic. After reading the full text of these papers, 73 articles were considered relevant and were included to extract the data for this review (Fig. 2).

### Data extraction

The studies were identified by a single investigator (BA), and the following data were double-extracted independently by two reviewers (VK and AS): first author, publication year, location or ethnicity of participants, sample size, mean age, study design, SNP position, name and reference SNP (rs) ID, genotype and allele distribution for vitamin D. Corresponding authors were contacted to provide any additional information where needed.

This review will look at the genes that function upstream and influence 25(OH)D synthesis (*DHCR7, CYP2R1)* and the genes that function downstream and play a role in 25(OH)D metabolism (*CYP24A1, CYP27B1, GC/DBP, VDR)* (Fig. 1). The following sections will focus on the SNPs in the vitamin D pathway-related genes and their associations with obesity traits and T2D.

## Vitamin D synthesis genes

Despite several studies that have examined the association between vitamin D deficiency and metabolic diseases [13–16], the literature is remarkably scarce in studies investigating the association of genes involved in the synthesis of 25(OH)D with metabolic diseases such as obesity and T2D.

### 7-Dehydrocholesterol Reductase (*DHCR7*)

7-Dehydrocholesterol reductase enzyme is encoded by the *DHCR7* gene and is located on chromosome 11q13.4. The main function of DHCR7 is to convert 7DHC to cholesterol [12]. 7DHC is a substrate of the enzyme DHCR7 and is the precursor of vitamin D (specifically vitamin D_3_). The conversion of 7DHC to vitamin D_3_ in the skin is facilitated by exposure to UVB light from the sun which causes the cleavage of the C (9–10) bond in 7DHC to form vitamin D_3_ [17]. The production of cholesterol from 7DHC in the skin reduces the availability of 7DHC for vitamin D synthesis, which limits vitamin D production [18].

### Cytochrome P450 Family 2 Subfamily R member 1 / 25-Hydroxylase (*CYP2R1*)

The *CYP2R1* gene is located on chromosome 11q15.2. *CYP2R1* encodes 25-hydroxylase enzyme in the liver, which is the main enzyme responsible for the conversion of vitamin D_3_ and vitamin D_2_ to the main circulating form of vitamin D [25(OH)D] [19].

#### Obesity

Two cross-sectional studies investigated SNPs in the vitamin D synthesis-related genes; *DHCR7* SNP was examined in a small study (n = 323) of African ethnicity and *CYP2R1* SNP was studied in nearly 7,000 Chinese women [20, 21]. Nominal significant associations were reported with obesity traits in the Chinese study for *CYP2R1* rs10832313 polymorphism; however, this did not remain significant after correction for multiple testing [20] and no significant associations were reported for *DHCR7* SNP rs12785878 in the African population [21]. There are two notable large studies by Vimaleswaran et al. that examined SNPs from the *DHCR7* and *CYP2R1* vitamin D synthesis-related genes in relation to obesity using data from multiple Caucasian cohorts [22, 23]. One study was a bi-directional Mendelian Randomization study (n = 42,024) which showed that 10% genetically higher BMI was associated with 4.2% lower concentrations of 25(OH)D but no significant effect of vitamin D allelic scores on obesity was reported [22]. The other study was a genetic association analysis that analyzed 5,224 participants from the 1958 British birth cohort (1958BC) and 123,865 individuals from the GIANT (Genetic Investigation of Anthropometric Traits) consortium. None of the vitamin D synthesis SNPs was significantly associated with obesity traits [23]. Although the number of studies that investigated the association between vitamin D synthesis genes and obesity were small, the lack of association found in the two very large meta-analysis studies [22, 23] does suggest that the vitamin D synthesis-related gene polymorphisms may not be a contributing factor to the development of obesity. However, further studies with large number of samples are required to confirm the role of these SNPs in obesity and its related traits.

#### Type 2 diabetes

Studies using vitamin D synthesis-related gene polymorphisms suggest an association with low serum 25(OH)D concentrations and increased diabetes risk [24]. A recent prospective observational study in Italians (n = 2,163) demonstrated an association between *DHCR7* (rs12785878) and 25(OH)D concentrations (P = 1 × 10^**− 4**^) in T2D patients [25]. Furthermore, in a recent large Mendelian Randomization meta-analysis of 10 studies from European and Chinese populations (n = 58,312 cases and 370,000 controls), the allelic score of two SNPs from the vitamin D synthesis-related genes, *DHCR7* (rs12785878) and *CYP2R1* (rs10741657), were shown to be significantly associated with lower risk of T2D (P = .01), where a 25 nmol/l higher 25(OH)D concentration was associated with a 14% lower risk of diabetes [26]. A Mendelian Randomization analysis in 96,423 Danish individuals examined four genetic polymorphisms in the *DHCR7* and *CYP2R1* genes in relation to T2D [24], where the *DHCR7* allele score (rs11234027 + rs7944926) showed a significant association with increased risk of T2D (P for trend = 0.04); but there were no significant associations between *CYP2R1* SNPs or allele scores and risk of diabetes [24]. In the Chinese Han population (n = 794), the ‘G’ allele carriers of the *CYP2R1* SNPs, rs10766197 and rs1993116, had 1.64 and 1.76 times increased risk of developing T2D compared with ‘AA’ homozygotes, respectively (P = .024 and P = .048, respectively) [27]. However, the studies in 53,088 Germans and 4,877 Norwegians failed to show an association of SNPs in the *DHCR7* (rs12785878, rs3829251, rs3794060) and *CYP2R1* (rs10741657) genes with T2D [28, 29]. Although there is inconsistency in the results across various studies, a recent large Mendelian Randomization meta-analysis (n = 428,312) [26] has provided evidence that genetically instrumented higher 25(OH)D has a protective effect against diabetes risk. However, more studies are needed to confirm this finding and understand the functional significance of vitamin D synthesis-related genes in T2D.

## Vitamin D metabolism genes

Several studies have examined the association of vitamin D metabolism-related SNPs with metabolic diseases; however, majority of the studies have been restricted to the *VDR* SNPs and only a few studies have investigated the association of genes involved in activation, catabolism and transport of 25(OH)D with metabolic diseases. Hence, *VDR*–related SNPs are discussed in a separate section.

### I. Activation, Catabolism, and Transport Genes

#### Cytochrome P450 Family 27 Subfamily B member 1 / 1α-Hydroxylase *(CYP27B1)*

The *CYP27B1* gene is located on chromosome 12q14.1. The activating enzyme 1α-hydroxylase is encoded by the *CYP27B1* gene in the kidney where 25(OH)D is converted to the active 1α,25(OH)_2_D which binds to the *VDR* to perform its biological functions [30, 31].

#### Cytochrome P450 Family 24 Subfamily A member 1 / 24-Hydroxylase *(CYP24A1)*

The *CYP24A1* gene, which is located on chromosome 20q13.2, codes for the vitamin D inactivating enzyme 24-hydroxlase. This enzyme controls the levels of vitamin D in blood serum by breaking down the active form to biliary excretory products and by reducing intestinal absorption of calcium and phosphate. Mutations in the gene have been shown to result in hypercalcemia and nephrolithiasis [32, 33].

#### Vitamin D Binding Protein *(DBP)* / Group-Specific Component *(GC)*.

The *DBP*/*GC* gene is located on chromosome 4q13.3. The *GC* gene encodes for vitamin D binding protein (DBP), which is a glycoprotein secreted by the liver, that binds to vitamin D and its metabolites from the gut and skin and transports them to target tissues and organs and, hence, factors affecting DBP levels can also affect vitamin D concentrations. Nearly 85% of serum 25(OH)D is bound to DBP and the remainder 15% binds to albumin. Approximately 0.4% of total 1α,25(OH)_2_D_3_ and 0.03% of total 25(OH)D_3_ exist in the unbound, free form in the serum of healthy individuals (excluding pregnant women) [34, 35].

#### Obesity

To date, there has been only one study in Europeans (n = 5,224) [23] that has investigated the association of *CYP27B1* SNPs, rs1048691 and rs10877012, with obesity and this study did not find any significant association of these SNPs with obesity-related outcomes. The same study in 5,224 Europeans also failed to show an association of 22 *CYP24A1* SNPs with obesity [23]. This is in line with another study in up to 700 Chinese women [20], which also did not show a significant association of the *CYP24A1* SNP rs2248359 with obesity traits after correction for multiple testing. For the *GC* gene, while a study in 5,224 participants from the 1958 British Birth Cohort failed to show an association of the 13 SNPs in the *GC* gene with obesity outcomes [23], studies in Caucasian nuclear families [36] (n = 1,837), Bahraini population [37] (n = 406) and African population [21] (n = 323) showed significant associations of SNPs in the *GC* gene with obesity-related outcomes. Based on the Quantitative Transmission Disequilibrium Test in the Caucasian nuclear family study, it was shown that the *GC* SNP rs17467825 increased percent fat mass (PFM) by 1.42 times and the haplotype ‘GAA’ in the *GC* gene increased PFM by 1.19 times [36]. In the African population, the ‘TT’ genotype of the *GC* SNP rs2298849 was associated with 1.76 times increased risk of overweight [21]. Furthermore, the vitamin D metabolism-related genes were also analysed as a risk score in a Mendelian Randomization study in 42,024 Caucasians and there was no significant causal effect of the vitamin D metabolism-risk score on obesity [22].

Only a few studies have focused on the genes involved in activation, catabolism and transport of 25OHD of which there has been only one study on activation gene *CYP27B1* in relation to obesity [23]. Three studies have examined the catabolism gene *CYP24A1* SNPs; however, none of them showed a significant association with obesity traits [20, 22, 23]. Five studies included SNPs from the transport gene *GC* [21–23, 36, 37], where only three studies showed significant associations with obesity [21, 36, 37]. Given that majority of the studies failed to find an association of these SNPs with obesity outcomes, it is quite unlikely for the SNPs in the *CYP27B1*, *CYP24A1* and *GC* to have a significant functional role in obesity-related metabolic pathways.

#### Type 2 diabetes

There have been only two studies [28, 38] that examined SNPs from vitamin D activating *CYP27B1* gene in relation to T2D and both the studies, the prospective case-cohort study in 53,088 Germans [28] and a cross-sectional study in 522 individuals from a Polish population [38], failed to show an association of *CYP27B1* SNPs, rs10877012 and rs184712, with T2D, respectively. Five studies have explored the association of SNPs in vitamin D catabolism *CYP24A1* gene with T2D [26, 28, 29, 39, 40]. Two Chinese case-control family-based studies (n = 1,560 & n = 1,556) examined *CYP24A1* SNPs rs2248359 and rs4809957 [39, 40]; while the study in 1,556 individuals showed no association of the SNP rs4809957 with T2D [40], the study in 1,560 individuals demonstrated an association of the SNP rs2248359 with T2D in women (P = .036) but not in men (P = .816) [39]. It was shown that the ‘T’ allele of the SNP rs2248359 was transmitted 1.39 times more in offspring of T2D participant compared to non-T2D participant (P = .035) suggesting that ‘T’ allele might be a risk factor for T2D.

Despite large sample size, three studies, one in Germans (n = 53,088) [28], the other in Norwegians (n = 4,877) [29], and a study in Chinese (n = 5,566) [26] failed to find an association between *CYP24A1* SNP rs6013897 and T2D. In the vitamin D transport gene, *GC*, significant associations were reported in Asians [41–43] but not in European populations such as Germans, Polish and Norwegians [26, 28, 29, 44]. However, a recent prospective observational study in Europeans recruited from Italian outpatient clinics did provide an evidence of association between *GC* (rs4588) and 25(OH)D concentrations (P = 1 × 10^− 6^) in T2D patients [25]. The *GC* SNPs, rs7041 (codon 416) and rs4588 (codon 420), showed a significant association with T2D in a Bangladeshi population (n = 211) [42]. The participants with Glu/Glu at codon 416 had 2.87 times increased risk of T2D and the participants with Lys/Lys genotype at codon 420 had 8.9 times increased risk of T2D. Furthermore, the combined allele score of these two SNPs, rs7041 and rs4588, was significantly associated with T2D in a case-control study in a Pakistani population (n = 330) [41]. In a meta-analysis of studies of Caucasians and Asians, there was no association in the overall analysis of SNPs at codon 416 and codon 420 with the risk of T2D, however, after stratification based on ethnicity, a significant association was found in Asians at codon 420, where the allele ‘Lys’ had a 1.49 times increased risk of T2D. In addition, a 1.36 times increased T2D risk was observed for those with ‘Asp/Asp’ genotype at codon 416 compared to those with ‘Glu/Asp’ and ‘Glu/Glu’ genotypes [43].

To date, most of the large studies failed to demonstrate a significant association of the SNPs in the *CYP27B1* and *CYP24A1* genes with T2D suggesting that these genes are unlikely to play a potential role in the pathogenesis of T2D. However, based on the published studies, genetic variants in *GC* gene may have an impact on T2D among Asians but not in Europeans, which could be due to the existence of genetic heterogeneity across the two ethnicities. The possible mechanism of action of GC in T2D could be mediated through the regulation of plasma calcium levels, which is known to regulate insulin synthesis and secretion, and through a direct action on pancreatic beta-cell function [45].

### II. The Vitamin D Receptor (*VDR*)

The *VDR* gene is located on chromosome 12q13.11. Vitamin D receptor, encoded by *VDR* gene, is a member of the nuclear receptor of transcription factors. The secosteroid 1α,25(OH)_2_D_3_, a natural ligand to *VDR*, enters the target cell and binds to its receptor. The 1α,25(OH)_2_D_3_-*VDR* complex heterodimerizes with the retinoid X receptor (*RXR*) and binds to the vitamin D response element (VDRE), a sequence of DNA nucleotides in the promoter region of the vitamin D regulated genes. The VDR/RXR-VDRE complex attracts coactivators and gene transcription is initiated to produce mRNA, which is then translated to the corresponding protein [46–48]. The *VDR* gene is predominantly expressed in kidneys, bones and the intestinal tract for bone homeostasis but further expression has been discovered in almost all human tissues and organs including adipose tissue and cells involved in the regulation of glucose metabolism, such as muscle and pancreatic cells [49]. Several functional *VDR* SNPs are known: BsmI, ApaI, Tru9I in intron 8, TaqI in exon 9 and FokI in exon 2. These genetic variants are named after their restriction enzyme sites [49]. Another *VDR* SNP is Cdx2, which is found in the promoter region [3]. Some of the SNPs in the *VDR* are restriction fragment length polymorphisms such as BsmI, ApaI and TaqI. *VDR* SNPs are closely linked to a microsatellite poly A repeat of variable length in the 3´UTR region which is thought to affect *VDR* translation and may affect mRNA stability [3, 50]. *VDR* SNPs such as BsmI, ApaI and TaqI and FokI are the most commonly studied genetic variants in association with non-skeletal outcomes [51]. Genetic variants in the *VDR* gene have also shown to contribute to the genetic susceptibility of T2D by modulating insulin secretion and affecting cellular insulin sensitivity [45]. Allelic differences in the *VDR* gene have also shown to be possible contributors to obesity through modulating adipocyte function and affecting adipocyte inflammation [45, 51, 52].

#### Obesity

##### BsmI SNP rs1544410

Thirteen studies have examined the association between the *VDR* BsmI SNP and obesity-related traits (Table 1), of which nine have reported a significant association in the Arab, Brazilian, Polish, French, Swedish, and Vietnamese populations (n = 140–891) [53–61]. In the Arab population, associations have been consistent in four studies (n = 198 – n = 891) [53–55, 60] suggesting that the presence of BsmI risk allele could be a risk factor for obesity in this ethnic group. Nevertheless, the sample sizes were relatively small; hence, larger studies are required in this population to confirm the risk of BsmI polymorphism on obesity.

**Table 1:** Studies that have investigated the association between vitamin D-related gene polymorphisms and obesity-related traits

Gene Symbol and Name	Chromosomal Location	SNP and nucleotide change (as per dbSNP)	Minor Allele Frequency	Study Design	Ethnicity and Sample Size	Age	Outcome Measure	Association P-Value	References
*DHCR7 / NADSYN1* 7-Dehydrocholesterol reductase / NAD Synthetase 1	11q13.4	rs12785878 G/A,T	T = 0.21	Cross-sectional	African n = 323	46 ± 12	Obesity	0.93 ^*^	Foucan et al. [21]
			G = 0.22–0.40^ψ^	Meta-analysis	Caucasians n = 42,024	31.16 ± 0.4–74.86 ± 2.9	BMI	0.78 ^*^	Vimaleswaran et al.[22]
*CYP2R1* Cytochrome P450 Family 2 Subfamily R Member 1 (25-Hydroxylase)	11p15.2	rs10832313 A/G	G = 0.05	Cross-sectional	Chinese Women n = 6922	25–70	BMI	0.02 ^α^ (not significant after multiple testing)	Dorjgochoo et al. [20]
		rs10741657 A/G	G = 0.31–0.43^ψ^	Meta-analysis	Caucasians n = 42,024	31.16 ± 0.4–74.86 ± 2.9	BMI	0.30 ^*^	Vimaleswaran et al.[22]
Synthesis Score *DHCR7 + CYP2R1*		rs12785878 + rs10741657		Meta-analysis	Caucasians n = 42,024	31.16 ± 0.4–74.86 ± 2.9	BMI	0.57 ^*^	Vimaleswaran et al.[22]
*CYP24A1* Cytochrome P450 Family 24 Subfamily A Member 1 (24-Hydroxylase)	20q13.2	rs2248359 C/T	T = 0.34	Cross-sectional	Chinese Women n = 6922	25–70	BMI	0.02 ^α^ (not significant after multiple testing)	Dorjgochoo et al. [20]
				Cross-sectional	British n = 5,224	45	BMI/WC/ WHR	0.26 ^α^/0.16 ^α^/ 0.48 ^α^	Vimaleswaran et al. [23]
				Cross-sectional	Europeans n = 123,865	30–79	BMI/WHR	0.29 ^α^/ 0.86 ^α^	Vimaleswaran et al. [23]
		rs6013897 T/A	A = 0.19–0.26^ψ^	Meta-analysis	Caucasian n = 42,024	31.16 ± 0.4–74.86 ± 2.9	BMI	0.61 ^*^	Vimaleswaran et al.[22]
*GC / VDBP* Group-Specific Component / Vitamin D Binding Protein	4q13.3	rs2282679 T/G	T = 0.30	Cross-sectional	Bahraini n = 406	34.07 ± 10.86	BMI	0.36 ^α^	Almesri et al. [37]
			G = 0.19–0.38^ψ^	Meta-analysis	Caucasian n = 42,024	31.16 ± 0.4–74.86 ± 2.9	BMI	0.91 ^*^	Vimaleswaran et al.[22]
			G = 0.07	Cross-sectional	African n = 323	46 ± 12	Obesity	0.20 ^*^	Foucan et al. [21]
		rs4588 (codon 420) G /A,T	G = 0.22	Cross-sectional	Bahraini n = 406	34.07 ± 10.86	BMI	0.43 ^α^	Almesri et al. [37]
				Cross-sectional	British n = 5,224	45	BMI/WC/ WHR	0.32 ^α^/0.72 ^α^/ 0.72 ^α^	Vimaleswaran et al. [23]
				Cross-sectional	Europeans n = 123,865	30–79	BMI/WHR	--/ --	Vimaleswaran et al. [23]
		rs7041 (codon 416) A/C,T	C = 0.46	Cross-sectional	Bahraini n = 406	34.07 ± 10.86	BMI	0.007 ^α^	Almesri et al. [37]
				Cross-sectional	British n = 5,224	45	BMI/WC/ WHR	0.89 ^α^/0.24 ^α^/ 1.00 ^α^	Vimaleswaran et al. [23]
				Cross-sectional	Europeans n = 123,865	30–79	BMI/WHR	0.66 ^α^/ 0.43 ^α^	Vimaleswaran et al. [23]
		rs2298849 A/G	A = 0.38	Cross-sectional	Bahraini n = 406	34.07 ± 10.86	BMI	0.993 ^α^	Almesri et al. [37]
			G = 0.41	Cross-sectional	African n = 323	46 ± 12	Obesity	0.04 ^*^	Foucan et al. [21]
				Cross-sectional	British n = 5,224	45	BMI/WC/ WHR	1.00 ^α^/1.00 ^α^/ 0.16 ^α^	Vimaleswaran et al. [23]
				Cross-sectional	Europeans n = 123,865	30–79	BMI/WHR	0.51 ^α^/ 0.97 ^α^	Vimaleswaran et al. [23]
		rs1491711 C/G	C = 0.33	Cross-sectional (family-based)	US Caucasian n = 1,873	47.49 ± 16.41	BMI/FM/ PFM	0.23 ^α^/0.30 ^α^/ 0.047 ^α^	Jiang et al. [36]
		rs17467825 A/G	G = 0.27	Cross-sectional (family-based)	US Caucasian n = 1,873	47.49 ± 16.41	BMI/FM/ PFM	0.048 ^α^/0.006 ^α^/ 0.001 ^α^	Jiang et al. [36]
		rs705117 C/T	C = 0.16	Cross-sectional (family-based)	US Caucasian n = 1,873	47.49 ± 16.41	BMI/FM/ PFM	0.32 ^α^/0.037 ^α^/ 0.11 ^α^	Jiang et al. [36]
				Cross-sectional	British n = 5,224	45	BMI/WC/ WHR	0.53 ^α^/0.78 ^α^/ 0.72 ^α^	Vimaleswaran et al. [23]
				Cross-sectional	Europeans n = 123,865	30–79	BMI/WHR	0.51 ^α^/ 0.65 ^α^	Vimaleswaran et al. [23]
		rs222042 G/A	A = 0.07	Cross-sectional (family-based)	US Caucasian n = 1,873	47.49 ± 16.41	BMI/FM/ PFM	> 0.05 ^α^/>0.05 ^α^/ > 0.05 ^α^	Jiang et al. [36]
		rs222040 G/A	G = 0.42	Cross-sectional (family-based)	US Caucasian n = 1,873	47.49 ± 16.41	BMI/FM/ PFM	> 0.05 ^α^/>0.05 ^α^/ > 0.05 ^α^	Jiang et al. [36]
		rs222035 T/G	T = 0.43	Cross-sectional (family-based)	US Caucasian n = 1,873	47.49 ± 16.41	BMI/FM/ PFM	> 0.05 ^α^/>0.05 ^α^/ > 0.05 ^α^	Jiang et al. [36]
		rs222003 C/A,G,T	C = 0.08	Cross-sectional (family-based)	US Caucasian n = 1,873	47.49 ± 16.41	BMI/FM/ PFM	> 0.05 ^α^/>0.05 ^α^/ > 0.05 ^α^	Jiang et al. [36]
		rs16846971 T/A	T = 0.002	Cross-sectional (family-based)	US Caucasian n = 1,873	47.49 ± 16.41	BMI/FM/ PFM	> 0.05 ^α^/>0.05 ^α^/ > 0.05 ^α^	Jiang et al. [36]
		rs222020 C/T	C = 0.16	Cross-sectional (family-based)	US Caucasian n = 1,873	47.49 ± 16.41	BMI/FM/PFM	> 0.05 ^α^/>0.05 ^α^/ > 0.05 ^α^	Jiang et al. [36]
				Cross-sectional	British n = 5,224	45	BMI/WC/ WHR	1.00 ^α^/1.00 ^α^/ 0.16 ^α^	Vimaleswaran et al. [23]
				Cross-sectional	Europeans n = 123,865	30–79	BMI/WHR	0.15 ^α^/ 0.44 ^α^	Vimaleswaran et al. [23]
		rs16847015 C/A,T	A = 0.04	Cross-sectional (family-based)	US Caucasian n = 1,873	47.49 ± 16.41	BMI/FM/PFM	> 0.05 ^α^ />0.05 ^α^/ > 0.05 ^α^	Jiang et al. [36]
		rs1352843 T/C	C = 0.12	Cross-sectional (family-based)	US Caucasian n = 1,873	47.49 ± 16.41	BMI/FM/PFM	> 0.05 ^α^/>0.05 ^α^/ > 0.05 ^α^	Jiang et al. [36]
		rs222029 G/A	G = 0.17	Cross-sectional (family-based)	US Caucasian n = 1,873	47.49 ± 16.41	BMI/FM/PFM	> 0.05 ^α^ />0.05 ^α^/ > 0.05 ^α^	Jiang et al. [36]
		rs3733359 G/A	A = 0.05	Cross-sectional (family-based)	US Caucasian n = 1,873	47.49 ± 16.41	BMI/FM/PFM	> 0.05 ^α^/>0.05 ^α^ / > 0.05 ^α^	Jiang et al. [36]
		rs16847036 A/G	G = 0.05	Cross-sectional (family-based)	US Caucasian n = 1,873	47.49 ± 16.41	BMI/FM/PFM	> 0.05 ^α^/>0.05 ^α^/ > 0.05 ^α^	Jiang et al. [36]
Synthesis Score *GC + CYP24A1*		rs2282679 + rs6013897		Meta-analysis	Caucasian n = 42,024	31.16 ± 0.4–74.86 ± 2.9	BMI	0.67 ^*^	Vimaleswaran et al.[22]
*VDR* Vitamin D Receptor	12q13.11	BsmI ^φ^ rs1544410 C/A,G,T (B/b)	b = 0.42	Case-control	Saudi n = 570	45.9 ± 14.5	Obesity/BMI/ WC	0.04 ^*^/0.08 ^γ^/ 0.57 ^γ^	Al-Daghri et al. [53]
			b = 0.40	Case-control	Saudi n = 891	39.6 ± 12.8	Obesity	0.028 ^β^	Al-Daghri et al. [54]
			b = 0.28	Case-control	Saudi Men n = 300	27.25 ± 4.22	Obesity/BMI	0.04 ^β^/0.02 ^α^	Al-Hazmi et al. [55]
			b = 0.37	Case-control	Czechs n = 882	48.3 ± 14.2	Obesity/WC/ SSFT/TBF	0.65 ^*^/0.055 ^α^/ 0.71 ^α^ /0.20 ^α^	Bienertova-Vasku et al. [62]
			b = 0.37	Cross-sectional	Spanish n = 701	20.41 ± 2.48	BMI/FM/ PFM/VFL	0.87 ^α^/0.86 ^α^ / 0.90 ^α^/0.93 ^α^	Correa-Rodriguez et al. [63]
			b = 0.39	Cross-sectional	Brazilian n = 319	10.6 ± 1.4	BMI/BFM	0.03 ^α^/0.24 ^α^	Ferrarezi et al. [57]
			B = 0.41	Cross-sectional	Polish Men n = 176	51.99 ± 10.73	BM/BMI/ WHR/ WC	0.23 ^α^/0.048 ^α^/ 0.75 ^α^/0.03 ^α^	Filus et al. [58]
			B = 0.44	Cross-sectional	Arabs n = 198	21 ± 9	WC/ BMI/ WHR/PBF	0.08 ^δ^/ 0.04 ^δ^/ 0.1 ^δ^/0.1 ^δ^	Hasan et al. [60]
			b = 0.19	Cross-sectional	Malaysian n = 941	13	Obesity/Wt./ BMI/WC/ WHR/PBF	0.40 ^*^/0.18 ^α^/ 0.26 ^α^/0.16 ^α^/ 0.69 ^α^ /0.31 ^α^	Rahmadhani et al. [50]
			B = 0.37	Cross-sectional	Polish Postmenopausal Women n = 351	55.43 ± 2.75	BMI/WC/ PTF/PVD	0.90 ^α^/ 0.86 ^α^/ 0.76 ^α^/ 0.92 ^α^	Tworowska-Bardzinska et al. [64]
			B = 0.39	Case-control	French n = 452	61.5 ± 14	BMI/PO	0.01 ^γ^/ 0.02 ^γ^	Ye et al. [61]
			B = 0.09	Cross-sectional	Vietnamese Postmenopausal Women n = 140	55.6 ± 3.8	Obesity	0.039 ^*^	Binh et al. [56]
			B = 0.38	Cross-sectional	Swedish n = 153	29.6 ± 5.9	BMI/FM	0.09 ^γ^/0.049 ^γ^	Grundberg et al. [59]
		ApaI ^φ^ rs7975232 C/A (A/a)	a = 0.37	Case-control	Saudi n = 570	45.9 ± 14.5	Obesity/BMI/ WC	0.27^*^/0.18 ^γ^/ 0.93 ^γ^	Al-Daghri et al. [53]
			a = 0.38	Case-control	Saudi n = 891	39.6 ± 12.8	Obesity	0.10 ^α^	Al-Daghri et al. [54]
			a = 0.41	Case-control	Saudi Men n = 300	27.25 ± 4.22	Obesity/BMI	0.32 ^α^/0.42 ^α^	Al-Hazmi et al. [55]
			a = 0.49	Case-control	Czechs n = 882	48.3 ± 14.2	Obesity/WC/ SSFT/TBF	0.2 ^*^/0.007 ^α^/ 0.31 ^α^/0.56 ^α^	Bienertova-Vasku et al. [62]
			A = 0.36	Case-control	Chinese Han n = 529	54.38 ± 11.08	Obesity	0.21 ^β^	Fan et al. [65]
			a = 0.41	Cross-sectional	Brazilian n = 319	10.6 ± 1.4	BMI/BFM	> 0.05 ^α^/>0.05 ^α^	Ferrarezi et al. [57]
			A = 0.28	Cross-sectional (family-based)	Chinese 415 sons	30.4 ± 6.1	BMI/ FM/PFM	0.99 ^α^/0.83 ^α^/ 0.57 ^α^/0.38 ^α^	Gu et al. [66]
			a = 0.40	Case-control	Mexican n = 250	47.3 ± 7.8	BMI	0.36 ^β^	Rivera-Leon et al. [67]
			a = 0.46	Case-control	French n = 452	61.5 ± 14	BMI/PO	0.09 ^γ^/0.09 ^γ^	Ye et al. [61]
			A = 0.33	Cross-sectional	Vietnamese Postmenopausal Women n = 140	55.6 ± 3.8	Obesity	0.036 ^*^	Binh et al. [56]
			A = 0.28	Cross-sectional	Chinese Postmenopausal Women n = 260	57.9	BMI	0.049 ^ε^	Xu et al. [68]
			a = 0.25	Cross-sectional	Chinese Han n = 517	18–90	BMI/WC/ PBF/TSFT	> 0.05 ^*^/>0.05 ^*^/ 0.02 ^α^/<0.001 ^α^	Shen et al. [69]
		TaqI ^φ^ rs731236 A/G (T/t)	t = 0.44	Case-control	Saudi n = 570	45.9 ± 14.5	Obesity/BMI/ WC	0.32 ^*^/0.26 ^γ^/ 0.94 ^γ^	Al-Daghri et al. [53]
			t = 0.41	Case-control	Saudi n = 891	39.6 ± 12.8	Obesity	0.009 ^α^	Al-Daghri et al. [54]
			t = 0.42	Case-control	Saudi Men n = 300	27.25 ± 4.22	Obesity/BMI	0.04 ^α^ /0.048 ^α^	Al-Hazmi et al. [55]
			t = 0.35	Cross-sectional	Bahraini n = 406	34.07 ± 10.86	BMI	0.98 ^α^	Almesri et al. [37]
			t = 0.37	Case-control	Czechs n = 882	48.3 ± 14.2	Obesity/WC/ SSFT/TBF	0.034 ^*^/0.035 ^α^/ 0.35 ^α^/0.88 ^α^	Bienertova-Vasku et al. [62]
			t = 0.36	Cross-sectional	Spanish n = 701	20.41 ± 2.48	BMI/FM/ PFM/VFL	0.90 ^α^/0.83 ^α^/ 0.88 ^α^ /0.93 ^α^	Correa-Rodriguez et al. [63]
			t = 0.10	Case-control	Chinese Han n = 529	54.38 ± 11.08	Obesity	< 0.001 ^β^	Fan et al. [65]
			t = 0.40	Cross-sectional	Brazilian n = 319	10.6 ± 1.4	BMI/BFM	> 0.05 ^α^/>0.05 ^α^	Ferrarezi et al. [57]
			t = 0.39	Cross-sectional	Arabs n = 198	21 ± 9	WC/ BMI/ WHR/PBF	0.55 ^δ^/ 0.58 ^δ^/ 0.9 ^δ^/0.55 ^δ^	Hasan et al. [60]
			t = 0.44	Case-control	Mexican n = 250	47.3 ± 7.8	BMI	0.80 ^β^	Rivera-Leon et al. [67]
			t = 0.39	Case-control	Greek n = 184	68.23 ± 8.99	Obesity/Waist	0.019 ^β^/0.87 ^α^	Vasilopoulos et al. [70]
			t = 0.39	Case-control	French n = 452	61.5 ± 14	BMI/PO	0.017 ^γ^/0.015 ^γ^	Ye et al. [61]
			t = 0.06	Cross-sectional	Vietnamese Postmenopausal Women n = 140	55.6 ± 3.8	Obesity	0.12 ^*^	Binh et al. [56]
				Cross-sectional	British n = 5,224	45	BMI/WC/ WHR	0.39 ^α^/0.70 ^α^/ 0.72 ^α^	Vimaleswaran et al. [23]
				Cross-sectional	Europeans n = 123,865	30–79	BMI/WHR	0.10 ^α^/ --	Vimaleswaran et al. [23]
		FokI ^φ^ rs2228570 A/C,G,T (F/f)	F = 0.27	Case-control	Saudi n = 570	45.9 ± 14.5	Obesity/BMI/ WC	0.23 ^*^/0.42 ^γ^/ 0.08 ^γ^	Al-Daghri et al. [53]
			F = 0.43	Case-control	Czechs n = 882	48.3 ± 14.2	Obesity/WC/ SSFT/TBF	0.055 ^*^/0.06 ^α^/ 0.046 ^α^/0.003 ^α^	Bienertova-Vasku et al. [62]
			f = 0.37	Cross-sectional	Spanish n = 701	20.41 ± 2.48	BMI/FM/PFM/ VFL	0.07 ^α^/0.34 ^α^/ 0.02 ^α^/0.43 ^α^	Correa-Rodriguez et al. [63]
			f = 0.35	Case-control	Chinese Han n = 529	54.38 ± 11.08	Obesity	0.36 ^β^	Fan et al. [65]
			f = 0.39	Cross-sectional	Polish men n = 176	51.99 ± 10.73	BM/BMI/ WHR/ WC	0.37 ^α^/0.87 ^α^/ 0.52 ^α^/0.47 ^α^	Filus et al. [58]
			f = 0.48	Cross-sectional (family-based)	Chinese n = 415 sons	30.4 ± 6.1	BMI/FM/ PFM	0.42 ^α^/0.24 ^α^/ 0.05 ^α^	Gu et al. [66]
			f = 0.23	Cross-sectional	Arabs n = 198	21 ± 9	WC/ BMI/ WHR/PBF	0.35 ^δ^/ 0.68 ^δ^/ 0.86 ^δ^/0.66 ^δ^	Hasan et al. [60]
			f = 0.46	Cross-sectional	Vietnamese Postmenopausal Women n = 140	55.6 ± 3.8	Obesity	0.15 ^*^	Binh et al. [56]
			f = 0.38	Cross-sectional	Caucasians Men n = 302	72.8 ± 0.8	BMI/FM/ PBF	0.01 ^ε^ /0.07 ^ε^ / 0.27 ^ε^	Roth et al. [71]
			F = 0.38	Cross-sectional	American Caucasian Women n = 1,773	57.2	BMI/WC/AH	0.43 ^α^ /0.67 ^α^ / 0.27 ^α^	Ochs-Balcom et al. [72]
			f = 0.47	Cross-sectional	Chinese Han n = 517	18–90	BMI/WC/ PBF/TSFT	> 0.05 ^*^/>0.05 ^*^/ 0.007 ^α^ /0.05 ^α^	Shen et al. [69]
		Cdx2 rs11568820 C/T	T = 0.48	Cross-sectional (family-based)	Chinese 415 sons	30.4 ± 6.1	BMI/FM/ PFM	0.006 ^α^/0.004 ^α^/ 0.002 ^α^	Gu et al. [66]
			T = 0.20	Cross-sectional	American Caucasian Women n = 1,773	57.2	BMI/WC/AH	0.09 ^α^/0.03 ^α^/ 0.05 ^α^ (WC and AH did not remain significant after Bonferroni correction)	Ochs-Balcom et al. [72]
				Cross-sectional	British n = 5,224	45	BMI/WC/ WHR	0.16 ^α^/0.31 ^α^/ 0.08 ^α^	Vimaleswaran et al. [23]
				Cross-sectional	Europeans n = 123,865	30–79	BMI/WHR	0.57 ^α^/ 0.72 ^α^	Vimaleswaran et al. [23]
		EcoRV rs4516035 T/C	C = 0.46	Case-control	Czechs n = 882	48.3 ± 14.2	Obesity/WC/ SSFT/TBF	0.67 ^*^/0.49 ^α^/ 0.02 ^α^ /0.39 ^α^	Bienertova-Vasku et al. [62]
				Cross-sectional	British n = 5,224	45	BMI/WC/ WHR	0.39 ^α^/0.49 ^α^/ 0.48 ^α^	Vimaleswaran et al. [23]
				Cross-sectional	Europeans n = 123,865	30–79	BMI/WHR	0.19 ^α^/ 0.11 ^α^	Vimaleswaran et al. [23]
		BglI rs739837 G/C,T	G = 0.47	Cross-sectional	American Caucasian Women n = 1,773	57.2	BMI/WC/AH	0.20 ^α^/0.16 ^α^/ 0.03 ^α^	Ochs-Balcom et al. [72]
			G = 0.46	Cross-sectional	British n = 5,160	45	Obesity/BMI/ WC/WHR	0.83 ^*^/0.43 ^α^/ 0.84 ^α^/0.30 ^α^	Vimaleswaran et al. [73]
				Cross-sectional	British n = 5,224	45	BMI/WC/ WHR	0.26 ^α^/0.29 ^α^/ 0.72 ^α^	Vimaleswaran et al. [23]
				Cross-sectional	Europeans n = 123,865	30–79	BMI/WHR	0.94 ^α^/ 0.28 ^α^	Vimaleswaran et al. [23]
		Tru9I rs757343 C/T	T = 0.13	Case-control	French n = 452	61.5 ± 14	BMI/PO	0.15 ^γ^/0.49 ^γ^	Ye et al. [61]
		rs1540339 C/T	T = 0.37	Cross-sectional	American Caucasian Women n = 1,773	57.2	BMI/WC/AH	0.56 ^α^/0.23 ^α^/ 0.24 ^α^	Ochs-Balcom et al. [72]
				Cross-sectional	British n = 5,224	45	BMI/WC/ WHR	0.48 ^α^/0.48 ^α^/ 0.16 ^α^	Vimaleswaran et al. [23]
				Cross-sectional	Europeans n = 123,865	30–79	BMI/WHR	0.25 ^α^/ 0.54 ^α^	Vimaleswaran et al. [23]
		rs2239179 T/C	C = 0.43	Cross-sectional	American Caucasian Women n = 1,773	57.2	BMI/WC/AH	0.10 ^α^/0.04 ^α^/ 0.02 ^α^ (WC and AH did not remain significant after Bonferroni correction)	Ochs-Balcom et al. [72]
			C = 0.44	Cross-sectional	British n = 5,160	45	Obesity/BMI/ WC/WHR	0.83 ^*^/0.23 ^α^/ 0.42 ^α^/0.63 ^α^	Vimaleswaran et al. [73]
			G = 0.22	Cross-sectional	Chinese Han n = 517	18–90	BMI/WC/ PBF/TSFT	> 0.05 ^*^/>0.05 ^*^/ 0.56 ^α^/0.001 ^α^	Shen et al. [69]
				Cross-sectional	British n = 5,224	45	BMI/WC/ WHR	0.48 ^α^/0.70 ^α^/ 0.29 ^α^	Vimaleswaran et al. [23]
				Cross-sectional	Europeans n = 123,865	30–79	BMI/WHR	0.47 ^α^/ 0.64 ^α^	Vimaleswaran et al. [23]
		rs12721377 T/C	T = 0.08	Cross-sectional	Bahraini n = 406	34.07 ± 10.86	BMI	0.32 ^α^	Almesri et al. [37]
		rs2189480 G/T	G = 0.34	Cross-sectional	Chinese Han n = 517	18–90	BMI/WC/ PBF/TSFT	> 0.05 ^*^/>0.05 ^*^/ 0.15 ^α^/0.07 ^α^	Shen et al. [69]
				Cross-sectional	British n = 5,224	45	BMI/WC/ WHR	1.00 ^α^/0.11 ^α^/ 0.08 ^α^	Vimaleswaran et al. [23]
				Cross-sectional	Europeans n = 123,865	30–79	BMI/WHR	0.61^α^/ 0.70 ^α^	Vimaleswaran et al. [23]
		rs3819545 A/G	G = 0.39	Cross-sectional	American Caucasian Women n = 1,773	57.2	BMI/WC/AH	0.04 ^α^/0.02 ^α^/ 0.05 ^α^	Ochs-Balcom et al. [72]
				Cross-sectional	British n = 5,224	45	BMI/WC/ WHR	0.67 ^α^/0.59 ^α^/ 0.48 ^α^	Vimaleswaran et al. [23]
				Cross-sectional	Europeans n = 123,865	30–79	BMI/WHR	0.65 ^α^/ 0.55 ^α^	Vimaleswaran et al. [23]
		rs3782905 G/C	G = 0.33	Cross-sectional	American Caucasian Women n = 1,773	57.2	BMI/WC/AH	0.001 ^α^/0.001 ^α^/ 0.001 ^α^	Ochs-Balcom et al. [72]
		rs2239186 A/C,G	G = 0.21	Cross-sectional	American Caucasian Women n = 1,773	57.2	BMI/WC/AH	0.23 ^α^/0.07 ^α^/ 0.58 ^α^	Ochs-Balcom et al. [72]
				Cross-sectional	British n = 5,224	45	BMI/WC/ WHR	0.64 ^α^/0.37 ^α^/ 0.29 ^α^	Vimaleswaran et al. [23]
				Cross-sectional	Europeans n = 123,865	30–79	BMI/WHR	0.73 ^α^/ 0.85 ^α^	Vimaleswaran et al. [23]
		rs2853564 G/A	G = 0.39	Cross-sectional	American Caucasian Women n = 1,773	57.2	BMI/WC/AH	0.84 ^α^/0.70 ^α^/ 0.19 ^α^	Ochs-Balcom et al. [72]
				Cross-sectional	British n = 5,224	45	BMI/WC/ WHR	0.12 ^α^/0.22 ^α^/ 0.48 ^α^	Vimaleswaran et al. [23]
				Cross-sectional	Europeans n = 123,865	30–79	BMI/WHR	0.09 ^α^/ 0.70 ^α^	Vimaleswaran et al. [23]
		rs4760648 C/A,G,T	T = 0.42	Cross-sectional	American Caucasian Women n = 1,773	57.2	BMI/WC/AH	0.30 ^α^/0.16 ^α^/ 0.04 ^α^	Ochs-Balcom et al. [72]
				Cross-sectional	British n = 5,224	45	BMI/WC/ WHR	1.00 ^α^/0.98 ^α^/ 0.16 ^α^	Vimaleswaran et al. [23]
				Cross-sectional	Europeans n = 123,865	30–79	BMI/WHR	0.09 ^α^/ 0.58 ^α^	Vimaleswaran et al. [23]
		rs3890734 G/A	A = 0.33	Cross-sectional	American Caucasian Women n = 1,773	57.2	BMI/WC/AH	0.53 ^α^/0.36 ^α^/ 0.08 ^α^	Ochs-Balcom et al. [72]
		rs7136534 C/T	T = 0.24	Cross-sectional	American Caucasian Women n = 1,773	57.2	BMI/WC/AH	0.07 ^α^/0.07 ^α^/ 0.11 ^α^	Ochs-Balcom et al. [72]
				Cross-sectional	British n = 5,224	45	BMI/WC/ WHR	0.21 ^α^/0.29 ^α^/ 0.08 ^α^	Vimaleswaran et al. [23]
				Cross-sectional	Europeans n = 123,865	30–79	BMI/WHR	0.44 ^α^/ 0.77 ^α^	Vimaleswaran et al. [23]
		rs10783210 T/A,G	T = 0.36	Cross-sectional	American Women n = 1,773	57.2	BMI/WC/AH	0.19 ^α^ /0.26 ^α^/ 0.66 ^α^	Ochs-Balcom et al. [72]
		rs7299460 C/T	T = 0.29	Cross-sectional	American Caucasian Women n = 1,773	57.2	BMI/WC/AH	0.10 ^α^/0.12 ^α^/ 0.09 ^α^	Ochs-Balcom et al. [72]
				Cross-sectional	British n = 5,224	45	BMI/WC/ WHR	1.00 ^α^ /0.08 ^α^ / 1.00 ^α^	Vimaleswaran et al. [23]
				Cross-sectional	Europeans n = 123,865	30–79	BMI/WHR	0.59 ^α^/ 0.91^α^	Vimaleswaran et al. [23]

Abbreviations: BMI: Body mass index, WC: Waist circumference, AH: Abdominal Height, SSFT: Sum of skin fold thickness, TBF: Total body fat, FM: Fat mass, PFM: Percentage fat mass, VF: Visceral fat, BM: Body mass, WHR: Waist hip ratio, PBF: Percent body fat, PTF: Percent total fat, PVD: Percent visceral deposit, TSFT: Triceps skinfold thickness

81 Tag SNPs that were investigated in the study by Vimaleswaran et al. [23] were not listed in this table because they showed no significant association with obesity traits and were not examined in other studies

^φ^Through literature reviewing we found that describing the genotype of *VDR* was very confusing. We decided on using the initial letter of the restriction enzyme to name the different alleles instead of using the nucleotide base letter for BsmI, ApaI, TaqI and FokI

^*^Logistic egression

^α^ Linear regression

^β^Chi square test

^γ^ANOVA

^δ^Kruskal-Wallis test

^ε^ANCOVA

^ψ^Indicates range value

##### ApaI SNP rs7975232

Twelve Studies have investigated the association between the *VDR* ApaI polymorphism and obesity traits (Table 1) and four of them have reported significant associations [56, 62, 68, 69] in the Chinese, Vietnamese and Czech populations (n = 140–882). Two studies in Asian postmenopausal women (n = 140; n = 260) reported significant associations with the ApaI variant (P = .036; P = .049, respectively) [56, 68], where the study in the postmenopausal Vietnamese women found that the ApaI risk allele ‘a’ had a 3 times increased risk of overweight and obesity [56]. Hence, ApaI risk allele may be an important factor predisposing individuals to adult onset obesity among Asian postmenopausal women; however, further large studies are warranted in men and women to validate the role of the ApaI variant in this group.

##### TaqI SNP rs731236

Fourteen studies have examined the association between the *VDR* TaqI polymorphism and obesity traits (Table 1), of which six have shown significant association in several populations including Saudi, Czech, Greek, French, and Chinese (n = 184–891) [54, 55, 61, 62, 65, 70]. In a case-control study in the Chinese Han population (n = 529), the TaqI polymorphism showed a strong association with obesity (P < .001) [65] where the ‘t’ allele was 2.67 times more prevalent in the obese group compared to the control group and the ‘tt’ genotype showed a 3.79 times increased risk of obesity. For the European and Arab populations, results have been largely inconsistent. Of the five studies in European population (n = 184–123,865), three reported an association between obesity traits and the TaqI polymorphism [61, 62, 70], while other studies failed to report a significant association (n = 701–123,865) [23, 63]. Similarly, in the Arab population, two studies reported an association of the TaqI polymorphism with obesity in Saudi individuals (n = 891; n = 300) [54, 55], where the minor ‘t’ allele was significantly more frequent in the obese group compared to the control group (P = .009; P = .041, respectively). But, three other studies in Arabs from Saudi, Bahrain, and UAE (n = 198–570) reported no significant association [37, 53, 60]. Although the results are inconsistent and conflicting, given that the majority of large studies failed to find an association, it is unlikely for the TaqI polymorphism to have a significant impact on obesity in Europeans and Arabs. However, due to existence of genetic heterogeneity, the polymorphism may have an effect on obesity in other ethnic groups such as Chinese population.

##### FokI SNP rs2228570

Twelve Studies have investigated the association between the *VDR* FokI polymorphism and obesity traits (Table 1) and only three of these have been consistent in reporting a significant association in Caucasian men (n = 302) and Czech (n = 517) and Chinese (n = 882) populations [62, 69, 71]. Studies in other ethnic groups such as Europeans, Asians, and Arabs (n = 140–1,773) failed to find an association of the FokI variant with obesity traits [53, 56, 58, 60, 61, 63, 65, 66, 72]. The overall evidence from these genetic epidemiological studies failed to support a consistent association of this polymorphism with obesity traits.

##### Cdx2 SNP rs11568820

Three Studies have investigated the association between the *VDR* Cdx2 SNP and obesity traits (Table 1). Two of these studies reported significant associations between Cdx2 SNP and obesity and its related traits [66, 72]. A significant association of Cdx2 SNP with waist circumference (WC) and abdominal height (AH) (P = .03; P = .05, respectively) was shown in a cross-sectional study in American Caucasian women (n = 1,773) [72]; however, the association did not remain significant after Bonferroni correction. On the other hand, a family-based study of 400 nuclear Chinese families (n = 1,215) [66] reported significant associations of Cdx2 SNP with body mass index (BMI) (P = .046), fat mass (FM) (P = .004) and PFM (P = .02). Furthermore, the analysis in 415 sons showed that those with ‘AA’ genotype had 5.4% higher BMI, 18.8% higher FM and 14.8% higher PFM compared to those with ‘AG’ genotype. Nonetheless, data from two large cohorts, the 1958 British Birth Cohort (n = 5,224) and the GIANT Consortium (n = 123,865), failed to find an association between the Cdx2 polymorphism and obesity-related traits [23]. Even though the results are inconsistent regarding the effect of the *VDR* Cdx2 polymorphism on obesity traits, majority of the large studies in Caucasians have failed to find significant associations and hence this polymorphism is unlikely to have an impact among the Caucasian population. However, such large studies in other ethnic groups are required to confirm the role of this polymorphism in obesity.

##### Other *VDR* SNPs

Four studies have investigated other *VDR* SNPs; of which, two have shown significant association [62, 72], while the other two failed to report a significant association with obesity traits [23, 69]. A study in American Caucasian women (n = 1,773) showed a significant association of five *VDR* SNPs (rs739837, rs2239179, rs3819545, rs3782905, and rs4760648) with obesity outcomes [72]. Another *VDR* SNP, EcoRV rs4516035 showed a significant association with sum of skin fold thickness (SSFT) (P = .02), where there was 7.7 times decrease in SSFT among those with the ‘GG’ genotype compared to those with ‘AA’ genotype in 882 Czech individuals [62]. Given that the studies have been conducted in small number of samples, large studies are required to further elucidate the role of these SNPs in obesity.

#### Type 2 Diabetes

##### BsmI SNP rs1544410

Twenty six studies have examined the association between the *VDR* BsmI polymorphism and T2D (Table 2), of which only seven have demonstrated a significant association in Arab, Indian, Chinese and German populations (n = 80–627) [74–80]. The remaining fifteen studies failed to show a significant association in populations of similar ethnicities (n = 57–4,563) [53, 61, 81–93]. The meta-analysis studies have also shown inconsistent findings, where, of the four meta-analyses in Asian and Caucasian populations (n = 2,608–6,274) [94–97], two of the studies (n = 4,578; n = 6,274) showed a marginal association between BsmI SNP and risk of T2D (P = .033; P = .038, respectively) [95, 96]. Despite several studies have been carried out in multiple ethnic groups, the association between the *VDR* BsmI variant and T2D is still questionable. It is possible that the effect of gene-lifestyle interactions might mask the genetic effect in some of the populations and hence studies focusing on gene-diet and gene-physical activity interactions are required to confirm this association.

**Table 2:** Studies that have investigated the association between vitamin D-related gene polymorphisms and diabetes-related traits

Gene Symbol and Name	Chromosomal Location	SNP and nucleotide change (as per dbSNP)	Minor Allele Frequency	Study Design	Ethnicity and Sample Size	Age	Outcome Measure	Association P-Value	References
*DHCR7 / NADSYN1* 7-Dehydrocholesterol reductase / NAD Synthetase 1	11q13.4	rs11234027 G/A	A = 0.20	Cross-sectional	Danish n = 96,423	20–100	T2D	0.11 ^*^	Afzal et al. [24]
		rs7944926 A/G	A = 0.32	Cross-sectional	Danish n = 96,423	20–100	T2D	0.03 ^*^	Afzal et al. [24]
		rs12785878 G/A,T	G = 0.27	Case-cohort	German n = 53,088	35–65	T2D	0.28 ^*^	Buijsse et al. [28]
			T = 0.46	Cross-sectional	Chinese n = 82,464 (sub-group)	51 ±10.6	T2D	> 0.05 ^*^	Lu et al. [26]
		rs3829251 G/A	A = 0.17	Case-cohort	German n = 53,088	35–65	T2D	0.22 ^*^	Buijsse et al. [28]
		rs3794060 C/T	C = 0.39	Case-cohort	Norwegians n = 4,877 (sub-group)	62.9 ± 12.45	T2D	> 0.05 ^*^	Jorde et al. [29]
*DHCR7* allele score		rs11234027 + rs7944926		Cross-sectional	Danish n = 96,423	20–100	T2D	0.04 ^*^	Afzal et al. [24]
*CYP2R1* Cytochrome P450 Family 2 Subfamily R Member 1 (25-Hydroxylase)	11p15.2	rs10741657 A/G	A = 0.42	Cross-sectional	Danish n = 96,423	20–100	T2D	0.78 ^*^	Afzal et al. [24]
			A = 0.38	Case-control	Chinese Han n = 794	59.53 ± 11.95	T2D	0.60 ^β^	Wang et al. [27]
			A = 0.39	Case-cohort	German n = 53,088	35–65	T2D	0.72 ^*^	Buijsse et al. [28]
			A = 0.42	Case-cohort	Norwegians n = 4,877 (sub-group)	62.9 ± 12.45	T2D	> 0.05 ^*^	Jorde et al. [29]
			A = 0.36	Cross-sectional	Chinese n = 82,464 (sub-group)	51 ± 10.6	T2D	> 0.05 ^*^	Lu et al. [26]
		rs12794714 G/A	A = 0.41	Cross-sectional	Danish n = 96,423	20–100	T2D	0.93 ^*^	Afzal et al. [24]
			A = 0.41	Case-control	Chinese Han n = 794	59.53 ± 11.95	T2D	0.09 ^β^	Wang et al. [27]
		rs10766197 G/A,C	A = 0.39	Case-control	Chinese Han n = 794	59.53 ± 11.95	T2D	0.024 ^β^	Wang et al. [27]
		rs1993116 A/G	A = 0.38	Case-control	Chinese Han n = 794	59.53 ± 11.95	T2D	0.048 ^β^	Wang et al. [27]
*CYP2R1* allele score		rs10741657 + rs12794714		Cross-sectional	Danish n = 96,423	20–100	T2D	0.84 ^*^	Afzal et al. [24]
Synthesis Score *DHCR7 + CYP2R1*		rs12785878 + rs10741657		Cross-sectional	Chinese n = 82,464 (sub-group)	51 ±10.6	T2D	> 0.05 ^*^	Lu et al. [26]
Synthesis Score *DHCR7 + CYP2R1*		rs12785878 + rs10741657		Meta-analysis	Chinese & European n = 428,904		T2D	0.01^*^	Lu et al. [26]
*CYP27B1* Cytochrome P450 Family 27 Subfamily B Member 1 (1α-Hydroxylase)	12q14.1•	rs10877012 G > C / G > T	T = 0.33	Case-cohort	German n = 53,088	35–65	T2D	0.77 ^*^	Buijsse et al. [28]
		rs184712 C/T	C = 0.18	Case-control	Polish n = 522	56.9 ± 11.8	T2D	0.65 ^β^	Malecki et al. [38]
	•	Intron 6	C = 0.34	Case-control	Polish n = 522	56.9 ± 11.8	T2D	0.67 ^β^	Malecki et al. [38]
*CYP24A1* Cytochrome P450 Family 24 Subfamily A Member 1 (24-Hydroxylase)	20q13.2•	rs6013897 T/A	A = 0.20	Case-cohort	German n = 53,088	35–65	T2D	0.56 ^*^	Buijsse et al. [28]
	•		A = 0.23	Case-cohort	Norwegians n = 4,877 (sub-group)	62.9 ± 12.45	T2D	> 0.05 ^*^	Jorde et al. [29]
	•		T = 0.84	Cross-sectional	Chinese n = 82,464 (sub-group)	51 ±10.6	T2D	> 0.05 ^*^	Lu et al. [26]
	•	rs4809957 A/G	A = 0.34	Case-control (family-based)	Chinese n = 1,556	59.4	T2D	0.65 ^β^	Yu et al. [40]
	•	rs2248359 C/T	T = 0.38	Case-control (family-based)	Chinese n = 1,560	50.77 ± 17.07	T2D	0.036 ^η^ (women)	Yu et al. [39]
*GC / VDBP* Group-Specific Component / Vitamin D Binding Protein	4q13.3•	rs2282679 T/G	G = 0.28	Case-cohort	German n = 53,088	35–65	T2D	0.99 ^*^	Buijsse et al. [28]
			G = 0.30	Cross-sectional	Chinese n = 82,464 (sub-group)	51 ±10.6	T2D	> 0.05 ^*^	Lu et al. [26]
	•	rs1155563 T/A,C	C = 0.28	Case-cohort	German n = 53,088	35–65	T2D	0.61 ^*^	Buijsse et al. [28]
	•	rs7041 (codon 416) A/C,T	C = 0.43	Case-control	Bangladeshi n = 211	39.7 ± 1.5	T2D	< 0.05 ^β^	Rahman et al. [42]
	•		G = 0.43	Case-control	Polish n = 393	56.4 ± 13.4	T2D	0.28 ^β^	Malecki et al. [44]
	•			Meta-analysis	Asian & Caucasian n = 2,073		T2D	> 0.05 ^*^	Wang et al. [43]
	•	rs4588 (codon 420) G/A,T	A = 0.27	Case-control	Bangladeshi n = 211	39.7 ± 1.5	T2D	< 0.01 ^β^	Rahman et al. [42]
	•		A = 0.30	Case-control	Polish n = 393	56.4 ± 13.4	T2D	0.52 ^β^	Malecki et al. [44]
	•			Meta-analysis	Asian & Caucasian n = 2,073		T2D	> 0.05 ^*^	Wang et al. [43]
	•			Meta-analysis	Asian n = 922 (sub-group)		T2D	< 0.05 ^*^	Wang et al. [43]
	•	rs2298850 G/C	C = 0.24	Case-cohort	Norwegians n = 4,877 (sub-group)	62.9 ± 12.45	T2D	> 0.05 ^*^	Jorde et al. [29]
*GC* allele score	•	rs7041 (codon 416) + rs4588 (codon 420)		Case-control	Pakistani n = 330	47.6 ± 9	T2D	< 0.05 ^*^	Iqbal et al. [41]
Synthesis & metabolism Score *DHCR7 + CYP2R1 + CYP24A1 + GC*	•	rs12785878 + rs10741657 + rs6013897 + rs2282679		Cross-sectional	Chinese n = 82,464 (sub-group)	51 ±10.6	T2D	> 0.05 ^*^	Lu et al. [26]
Synthesis & metabolism Score *DHCR7 + CYP2R1 + CYP24A1 + GC*	•	rs12785878 + rs10741657 + rs6013897 + rs2282679		Meta-analysis	Chinese & European n = 428,904		T2D	0.07 ^*^	Lu et al. [26]
*VDR* Vitamin D Receptor	12q13.11	BsmI ^φ^ rs1544410 C/A,G,T (B/b)	b = 0.41	Case-control	Saudi n = 627	47.8 ± 9.3	T2D	< 0.001 ^*^	Al-Daghri et al. [74]
			b = 0.42	Case-control	Saudi n = 570	45.9 ± 14.5	T2D/BG	0.11^*^/0.15 ^γ^	Al-Daghri et al. [53]
			b = 0.30	Case-control	Chilean n = 310	60–79	T2D	0.92 ^*^	Angel et al. [81]
			B = 0.41	Case-control	North Indian n = 260	49.32 ± 10.97	T2D	0.21 ^*^	Bid et al. [82]
			B = 0.45	Case-control	Bangladeshi n = 171	45.9± 10.3	ISI	0.23 ^*^	Hitman et al. [84]
			b = 0.24	Case-control	Egyptian n = 190	47.84 ± 6.75	T2D	0.95 ^β^	Mackawy et al. [85]
			b = 0.31	Case-control	Kashmiri n = 200	48.1 ± 9.9	T2D	0.0001 ^*^	Malik et al. [75]
			B = 0.48	Case-control	German n = 293	61.5 ± 9.9	T2D	0.002 ^β^	Ortlepp et al. [77]
			B = 0.36	Case-control	Polish n = 548	56.9 ± 12.2	T2D	0.29 ^β^	Malecki et al. [86]
			b = 0.47	Case-control	Emirati n = 355	54.1 ± 11.9	T2D	0.031 ^*^	Safar et al. [78]
			B = 0.42	Case-control	Caucasian n = 1,545	70.3 ± 8.9	T2D	> 0.05 ^β^	Oh et al. [87]
			b = 0.39	Case-control	Indian n = 60	49.05 ± 9.26	T2D	0.44 ^β^	Sarma et al. [88]
				Case-control	Czechs n = 234 (sub-group)	56 ± 9.88	T2D	> 0.05 ^*^	Vedralova et al. [91]
			Hui b = 0.12 Han b = 0.09	Case-control	Chinese Hui n = 269 Chinese Han n = 420		T2D	0.68 ^β^ 0.028 ^β^	Xu et al. [79]
			B = 0.39	Case-control	French n = 452	61.5 ± 14	T2D	0.96 ^β^	Ye et al. [61]
			b = 0.05	Case-control	Chinese Han n = 1,191	59.55 ± 11.96	T2D	0.84 ^β^	Yu et al. [92]
			b = 0.13	Case-control	Chinese Han n = 404	56.5 ± 10.7	T2D	0.015 ^β^	Zhang et al. [80]
				Case-control	Norwegians n = 4,563 (sub-group)	62.5 ± 12.5	T2D	> 0.05 ^ζ^	Zostautiene et al. [93]
			b = 0.34	Case-control	Indian n = 80	45.5 ± 11.5	T2D	< 0.05 ^*^	Mukhopadhyaya et al. [76]
			B = 0.46	Case-control	Indian Gujarati n = 57	> 45	T2D	> 0.05 ^β^	Shah et al. [89]
			B = 0.40	Case-control	Caucasians n = 187	23–83	T2D	> 0.05 ^β^	Speer et al. [90]
			b = 0.40	Case-control	Egyptian n = 100	51.74 ± 7.38	T2D	0.11 ^*^	Gendy et al. [83]
			B = 0.06 – b = 0.46^ψ^	Meta-analysis	Asian & Caucasian n = 2,608		T2D	0.23 ^*^	Zhu et al. [97]
			b = 0.05 – B = 0.42^ψ^	Meta-analysis	Caucasian & East Asian n = 4,578 (sub-group)	36.0 ± 4.9–71.7 ± 8.6	T2D	0.033^*^ (reported marginally significant)	Wang et al. [95]
			B = 0.10 – b = 0.46^ψ^	Meta-analysis	Asian & Caucasian n = 3,314 (sub-group)	40–62	T2D	0.70 ^*^	Li et al. [94]
			b = 0.05–0.42^ψ^	Meta-analysis	Chinese & European n = 6,274 (sub-group)	36.0 ± 4.9–71.7 ± 8.6	T2D	0.038^*^ (reported marginally significant)	Yu et al. [96]
		ApaI ^φ^ rs7975232 C/A (A/a)	a = 0.38	Case-control	Saudi n = 627	47.8 ± 9.3	T2D	0.05 ^*^ (not reported significant)	Al-Daghri et al. [74]
			a = 0.37	Case-control	Saudi n = 570	45.9 ± 14.5	T2D/BG	0.58 ^*^/0.42 ^γ^	Al-Daghri et al. [53]
			a = 0.39	Case-control	Turkish n = 241	56.6 ± 8.8	T2D/FPG /HbA1c	0.48 ^*^/0.11 ^α^/ 0.43 ^α^	Dilmec et al. [98]
			a = 0.42	Case-control	Bangladeshi n = 171	45.9± 10.3	ISI	0.006 ^*^	Hitman et al. [84]
			A = 0.49	Case-control	Polish n = 548	56.9 ± 12.2	T2D	0.33 ^β^	Malecki et al. [86]
			a = 0.40	Case-control	Mexican n = 250	47.3 ± 7.8	T2D/BG	0.98 ^β^/0.12 ^β^	Rivera-Leon et al. [67]
			a = 0.42	Case-control	Caucasian n = 1,545	70.3 ± 8.9	T2D	0.058 ^β^ (reported marginally significant)	Oh et al. [87]
				Case-control	Czechs n = 234 (sub-group)	56 ± 9.88	T2D	> 0.05 ^*^	Vedralova et al. [91]
			a = 0.46	Case-control	French n = 452	61.5 ± 14	T2D	0.20 ^β^	Ye et al. [61]
			a = 0.36	Case-control	Chinese Han n = 404	56.5 ± 10.7	T2D	0.39 ^β^	Zhang et al. [80]
				Case-control	Norwegians n = 4,563 (sub-group)	62.5 ± 12.5	T2D	> 0.05 ^ζ^	Zostautiene et al. [93]
			A = 0.47	Case-control	Indian Guadeloupe n = 189	51 ± 9.7	T2D	> 0.05 ^*^	Boullu-Sanchis et al. [99]
			A = 0.43	Case-control	Iranian n = 200	40 ± 8	T2D	0.54 ^β^	Nosratabadi et al. [100]
			A = 0.27–0.49^ψ^	Meta-analysis	Caucasian & East Asian n = 3,871 (sub-group)	36.0 ± 4.9–71.7 ± 8.6	T2D	0.98 ^*^	Wang et al. [95]
			A = 0.28–0.49^ψ^	Meta-analysis	Asian & Caucasian n = 3,381 (sub-group)	40–62	T2D	0.80 ^*^	Li et al. [94]
		TaqI ^φ^ rs731236 A/G (T/t)	t = 0.43	Case-control	Saudi n = 627	47.8 ± 9.3	T2D	0.07 ^*^	Al-Daghri et al. [74]
			t = 0.44	Case-control	Saudi n = 570	45.9 ± 14.5	T2D/BG	0.40 ^*^/0.70 ^γ^	Al-Daghri et al. [53]
			T = 0.38	Case-control	North Indian n = 260	49.32 ± 10.97	T2D	0.70 ^*^	Bid et al. [82]
			t = 0.36	Case-control	Turkish n = 241	56.6 ± 8.8	T2D/FPG /HbA1c	0.76 ^*^/0.11 ^α^/ 0.40 ^α^	Dilmec et al. [98]
			t = 0.32	Case-control	Bangladeshi n = 171	45.9± 10.3	ISI	0.06 ^*^	Hitman et al. [84]
			t = 0.32	Case-control	Brazilian n = 200	65.4 ± 8.18	T2D	1.00 ^*^	Maia et al. [101]
			t = 0.38	Case-control	Kashmiri n = 200	48.1 ± 9.9	T2D	0.67 ^*^	Malik et al. [75]
			t = 0.35	Case-control	Polish n = 548	56.9 ± 12.2	T2D	0.09 ^β^	Malecki et al. [86]
			t = 0.38	Case-control	Emirati n = 355	54.1 ± 11.9	T2D	0.84 ^*^	Safar et al. [78]
			t = 0.44	Case-control	Mexican n = 250	47.3 ± 7.8	T2D/Glucose	0.06 ^β^/0.74 ^β^	Rivera-Leon et al. [67]
			t = 0.39	Case-control	Caucasian n = 1,545	70.3 ± 8.9	T2D	> 0.05 ^β^	Oh et al. [87]
			t = 0.28	Case-control	Indian n = 60	49.05 ± 9.26	T2D	0.15 ^β^	Sarma et al. [88]
				Case-control	Czechs n = 234 (sub-group)	56 ± 9.88	T2D	> 0.05 ^*^	Vedralova et al. [91]
			Hui t = 0.07 Han t = 0.06	Case-control	Chinese Hui n = 269 Chinese Han n = 420		T2D	0.82 ^β^ 0.32 ^β^	Xu et al. [79]
			t = 0.39	Case-control	French n = 452	61.5 ± 14	T2D	0.94 ^β^	Ye et al. [61]
				Case-control	Norwegians n = 4,563 (sub-group)	62.5 ± 12.5	T2D	> 0.05 ^ζ^	Zostautiene et al. [93]
			T = 0.32	Case-control	Indian Guadeloupe n = 189	51 ± 9.7	T2D	> 0.05 ^*^	Boullu-Sanchis et al. [99]
			t = 0.39	Case-control	Indian n = 80	45 ± 11.5	T2D	< 0.05 ^*^	Mukhopadhyaya et al. [76]
			T = 0.36	Case-control	Iranian n = 200	40 ± 8	T2D	1.00 ^β^	Nosratabadi et al. [100]
			t = 0.33	Case-control	Turkish n = 200		T2D	> 0.05 ^β^	Vural et al. [102]
			t = 0.32	Case-control	Egyptian n = 100	51.74 ± 7.38	T2D	0.56 ^*^	Gendy et al. [83]
			T = 0.04 – t = 0.39^ψ^	Meta-analysis	Caucasian & East Asian n = 3,826 (sub-group)	36.0 ± 4.9–71.7 ± 8.6	T2D	0.53 ^*^	Wang et al. [95]
			T = 0.32 – t = 0.39^ψ^	Meta-analysis	Asian & Caucasian n = 3,435 (sub-group)	40–62	T2D	> 0.05 ^*^	Li et al. [94]
		FokI ^φ^ rs2228570 A/C,G,T (F/f)	f = 0.26	Case-control	Saudi n = 627	47.8 ± 9.3	T2D	0.14 ^*^	Al-Daghri et al. [74]
			F = 0.27	Case-control	Saudi n = 570	45.9 ± 14.5	T2D/BG	0.02 ^*^/0.30 ^γ^	Al-Daghri et al. [53]
			f = 0.47	Case-control	Chilean n = 310	60–79	T2D	0.04 ^*^	Angel et al. [81]
			f = 0.28	Case-control	North Indian n = 260	49.32 ± 10.97	T2D	0.10 ^*^	Bid et al. [82]
			f = 0.33	Case-control	Italians N = 1,713	49.5 ± 15.8	T2D	> 0.05 ^β^	Bertoccini et al. [103]
			f = 0.45	Case-control	Chinese Han n = 3,714	59.99 ± 9.90	T2D	0.24 ^*^	Jia et al. [104]
			f = 0.31	Case-control	Egyptian n = 190	47.84 ± 6.75	T2D	0.001 ^β^	Mackawy et al. [85]
			f = 0.36	Case-control	Brazilian n = 200	65.4 ± 8.18	T2D	0.13 ^*^	Maia et al. [101]
			f = 0.46	Case-control	Polish n = 548	56.9 ± 12.2	T2D	0.54 ^β^	Malecki et al. [86]
			f = 0.19	Case-control	Indian n = 60	49.05 ± 9.26	T2D	0.74 ^β^	Sarma et al. [88]
			f = 0.43	Case-control	Czechs n = 234 (sub-group)	56 ± 9.88	T2D	0.57 ^*^	Vedralova et al. [91]
			f = 0.29	Case-control	Emirati n = 355	54.1 ± 11.9	T2D	0.0007 ^*^	Safar et al. [78]
			F = 0.45	Case-control	Chinese Han n = 1,191	59.55 ± 11.96	T2D	0.69 ^β^	Yu et al. [92]
				Case-control	Norwegians n = 4,563 (sub-group)	62.5 ± 12.5	T2D	> 0.05 ^ζ^	Zostautiene et al. [93]
			f = 0.40	Case-control	Egyptian n = 100	51.74 ± 7.38	T2D	< 0.001 ^*^	Gendy et al. [83]
			f = 0.27–0.46^ψ^	Meta-analysis	Caucasian & East Asian n = 3,023 (sub-group)	36.0 ± 4.9–71.7 ± 8.6	T2D	< 0.001 ^*^	Wang et al. [95]
			f = 0.28–0.46^ψ^	Meta-analysis	Asian & Caucasian n = 2,070 (sub-group)	40–62	T2D	0.001 ^*^	Li et al. [94]
			f = 0.26–0.48^ψ^	Meta-analysis	Chinese & European n = 4,077 (sub-group)	36.0 ± 4.9–71.7 ± 8.6	T2D	< 0.001 ^*^	Yu et al. [96]
		Cdx2 rs11568820 C/T	T = 0.25	Case-control	Italian n = 1,788	47.85 ± 14.9	T2D	0.002 ^*^	Sentinelli et al. [105]
				Case-control	Norwegians n = 4,563 (sub-group)	62.5 ± 12.5	T2D	> 0.05 ^ζ^	Zostautiene et al. [93]
		BglI rs739837 G/C,T	C = 0.28	Case-control	Chinese Han n = 3,714	59.99 ± 9.9	T2D	0.002 ^*^	Jia et al. [104]
			Hui T = 0.31 Han T = 0.35	Case-control	Chinese Hui n = 269 Chinese Han n = 420		T2D	0.65 ^β^ 0.69 ^β^	Xu et al. [79]
			T = 0.26	Case-control	Chinese Han n = 1,191	59.55 ± 11.96	T2D	0.02 ^β^	Yu et al. [92]
			G = 0.46	Cross-sectional	European n = 5,160	45	T2D/HbA1c	0.18 ^*^/0.76 ^α^ /	Vimaleswaran et al. [73]
		Tru9I rs757343 C/T	Hui T = 0.21 Han T = 0.20	Case-control	Chinese Hui n = 269 Chinese Han n = 420		T2D	0.19 ^β^ 0.91 ^β^	Xu et al. [79]
			T = 0.13	Case-control	French n = 452	61.5 ± 14	T2D	0.21 ^β^	Ye et al. [61]
		rs2239179 T/C	C = 0.23	Case-control	Chinese Han n = 1,191	59.5 ± 11.9	T2D	0.049 ^β^	Yu et al. [92]
			C = 0.44	Cross-sectional	European n = 5,160	45	T2D/HbA1c	0.15 ^*^/0.57 ^α^/	Vimaleswaran et al. [73]
		rs7968585 C/G,T	C = 0.45	Case-control	Norwegians n = 4,563 (sub-group)	62.5 ± 12.5	T2D	0.044 ^ζ^	Zostautiene et al. [93]
		rs11574129 A/G	G = 0.18	Case-control	Chinese Han n = 3,714	59.99 ± 9.9	T2D	0.15 ^*^	Jia et al. [104]
		rs2189480 G/T	G = 0.36	Case-control	Chinese Henan n = 574	59.23 ± 12.31	T2D	< 0.003 ^*^	Han et al. [106]
		rs3847987 C/A	A = 0.20	Case-control	Chinese Henan n = 574	59.23 ± 12.31	T2D	0.03 ^*^	Han et al. [106]

Abbreviations: BG: Blood glucose, ISI: Insulin secretion index, FPG: Fasting plasma glucose, HbA1c: Haemoglobin A1c, FSG: Fasting serum glucose

^φ^ Through literature reviewing we found that describing the genotype of *VDR* was very confusing. We decided on using the initial letter of the restriction enzyme to name the different alleles instead of using the nucleotide base letter for BsmI, ApaI, TaqI and FokI

^*^Logistic egression

^α^ Linear regression

^β^ Chi square test

^γ^ ANOVA

^ζ^ Cox regression

^η^ Family-based association testing (FBAT)

^ψ^ Indicates range value

##### ApaI SNP rs7975232

None of the fifteen studies have demonstrated a significant association between ApaI polymorphism and risk of T2D (n = 171–4,563) (Table 2) including the two meta-analyses that investigated the association in up to 3,871 individuals [94, 95]. However, there was one study that reported a borderline association (P = .058) in a Caucasian US population (n = 1,545) [87]. Furthermore, a study in 171 Bangladeshi participants had shown a significant association (P = .006) between ApaI SNP and insulin secretion index (ISI) [84]. Based on these studies, it can be concluded that it is unlikely that the *VDR* ApaI SNP has a significant role in the development of T2D.

##### TaqI SNP rs731236

Despite twenty three studies have been carried out to explore the association between *VDR* TaqI polymorphism and T2D (Table 2), only one small study has reported a significant association in the Indian population (n = 80) where ‘t’ allele had a 1.5 times increased risk of T2D [76]. Even the two large meta-analyses in Caucasians and Asians (n up to 3,826) have failed to show an association [94, 95]. Given that majority of the large studies failed to demonstrate a significant association, the *VDR* TaqI SNP might not be a strong candidate for T2D.

##### FokI SNP rs2228570

To date, eighteen studies have examined the association between the *VDR* FokI SNP and T2D (Table 2); five studies in the Saudi, Emirati, Egyptian and the Chilean populations (n = 100–4,077) [53, 78, 81, 83, 85] and three meta-analyses (n = 2,070–4,077) in Asians and Caucasians [94–96] have reported significant associations. Given that large meta-analysis studies have confirmed the association of the SNP with T2D, the *VDR* FokI SNP might have an important role to play in T2D among Asians and Caucasians. Future meta-analyses should focus on other ethnic groups to identify the existence of genetic heterogeneity in the association between *VDR* FokI SNP and T2D.

##### Cdx2 SNP rs11568820

Only two studies have examined the association between the *VDR* Cdx2 SNP and T2D (Table 2); one in the Italian population (n = 1,788) where individuals with ‘AA’ genotype of the SNP had 1.43 times increased risk of T2D compared to those with ‘GG + GA’ genotypes (P = .002) [105] and the other one in the Norwegian populations (n = 4,563), which failed to show an association [93]. More studies in different ethnic groups are required to understand the role of the *VDR* Cdx2 SNP in T2D.

##### BglI SNP rs739837

Of the four studies that examined the association between the *VDR* BglI polymorphism and T2D (Table 2), two large case-control studies in the Chinese Han population (n = 1,191 and n = 3,714) reported a significant association of the BglI polymorphism with increased risk of T2D [92, 104]. However, there was no association between the BglI variant and risk of T2D in a smaller study in the Chinese Han (n = 420) and Chinese Hui (n = 269) populations [79] as well as no association was observed in the Caucasian population (1958 British Birth Cohort, n = 5,160) [73]. This inconsistency can be attributed to the sample size and the existence of genetic heterogeneity between the Caucasian and Chinese ethnic groups; but additional large studies are warranted to confirm or refute these findings.

##### Other *VDR* SNPs

A few studies have investigated other SNPs in the *VDR* gene; three studies reported a significant association of the *VDR* SNPs, rs2239179, rs7968585, rs2189480 and rs3847987, with T2D risk [92, 93, 106]. The two *VDR* SNPs, rs2189480 and rs3847987, were reported to have significant association (P = < 0.003 and P = .032, respectively) with T2D in a Chinese Henan population (n = 574) [106]. The *VDR* SNP rs2239179 was found to be significantly associated with increased risk of T2D (P = .049) in Chinese Han men who were above 55 years (n = 1,191) [92] and the *VDR* SNP rs7968585 showed a significant association with T2D (P = .044) in the Norwegian population (n = 4,563) [93]. These SNPs might have a functional importance in the pathogenesis of T2D, which needs to be further investigated and evaluated by large-scale studies in different populations.

## Conclusions

In summary, our review has pooled all available data related to the association of vitamin D pathway genes with metabolic diseases such as obesity and T2D and has identified 57 significant associations of vitamin D pathway genes with obesity and its related traits such as BMI, WC, FM, SSFT, and AH in SNPs from 5 genes in the vitamin D pathway. Of the 57 associations, only one was from the vitamin D synthesis-related genes (*DHCR7*, *CYP2R1)*. The vast majority of the associations (56 associations) were identified in the vitamin D metabolism-related genes (*CYP24A1, GC* and *VDR*) and, in particular, the *VDR* gene SNPs showed 48 significant associations with obesity outcomes. In addition, our review has identified 35 significant associations in relation to T2D in SNPs from 7 genes in the vitamin D pathway. A similar pattern of association was seen where only five significant associations were reported for vitamin D synthesis-related genes (*DHCR7* and *CYP2R1)* as compared to the vitamin D metabolism-related genes (*CYP24A1, GC* and *VDR*), where 30 significant associations were observed, and, in particular, 26 significant associations with T2D was seen for the *VDR* SNPs.

The vitamin D synthesis-related genes, *DHCR7* and *CYP2R1*, have not been adequately investigated in relation to obesity and T2D. There is a gap in the research pertaining to the effect of vitamin D synthesis-related gene polymorphisms on obesity and T2D. For vitamin D metabolism-related genes, the literature is still lacking in several ethnic groups, and available results are inconsistent. Understanding how genetics influence serum 25(OH)D levels is important for identifying persons at risk of vitamin D deficiency and improving the understanding of the observed association between vitamin D deficiency and several diseases. Large well-designed genetic association studies considering gene-environment interactions in multiple ethnic populations are necessary to improve the understanding of the role of vitamin D-related polymorphisms in metabolic diseases. Furthermore, functional characterization of the vitamin D-related SNPs is highly warranted to facilitate the understanding of the pathogenetic mechanisms of obesity and diabetes, which will provide the platform for developing strategies to prevent and treat metabolic diseases.

## Data Availability

The data generated from the literature search are included in the Tables 1 and 2.

## Abbreviations

T2D: Type 2 diabetes
SNP: Single nucleotide polymorphism
25(OH)D: 25 hydroxyvitamin D
1,25(OH)_2_D: 1,25 dihydroxyvitamin D
*DHCR7*: 7-dehydrocholesterol reductase
*CYP2R1*: 25-hydroxylase.
*CYP27B1*: 1α-hydroxylase
*CYP24A1*: 24-hydroxylase
*GC*: Group-specific component
*DBP*: Vitamin D binding protein
*VDR*: Vitamin D receptor
GWAS: Genome wide association studies
RXR: Retinoid X receptor
VDRE: Vitamin D receptor elements
BMI: Body mass index
PFM: Percentage fat mass
FM: Fat mass
WC: Waist circumference
AH: Abdominal Height
SSFT: Sum of skin fold thickness
TBF: Total body fat
VF: Visceral fat
BM: Body mass
WHR: Waist hip ratio
PBF: Percent body fat
PTF: Percent total fat
PVD: Percent visceral deposit
TSFT: Triceps skinfold thickness
BG: Blood glucose
ISI: Insulin secretion index
FPG: Fasting plasma glucose
HbA1c: Glycated haemoglobin
FSG: Fasting serum glucose
